# Evaluation of the Combined Effect of 2ME2 and ^60^Co on the Inducement of DNA Damage by IUdR in a Spheroid Model of the U87MG Glioblastoma Cancer Cell Line Using Alkaline Comet Assay

**Published:** 2011-08-24

**Authors:** Samideh Khoei, Sara Delfan, Ali Neshasteh-Riz, Seyed Rabi Mahdavi

**Affiliations:** 1. Medical Physics Department, School of Medical Basic Sciences, Tehran University of Medical Sciences, Tehran, Iran; 2. Cellular and Molecular Research Center, Tehran University of Medical Sciences, Tehran, Iran; 3. Radiology Department, College of Allied Medicine, Tehran University of Medical Sciences, Tehran, Iran

**Keywords:** Iododeoxyuridinre, DNA Damage, HIF-1Alpha, 2-Methoxyestradiol, Comet

## Abstract

**Objective:**

In this study, we investigated the combined effect of 2-Methoxyestradiol (2ME2) and ^60^Co on the cytogenetic damage of iododeoxyuridine (IUdR) in the spheroid model of U87MG glioblastoma cancer cell lines by alkaline comet assay.

**Materials and Methods:**

U87MG cells were cultured as spheroids with diameters of 350 µm. As control, the spheroids of one plate were not treated. Other cultures were pretreated with 2ME2 (250 µM) for one volume doubling time (1 VDT). After this time, the subsequent treatments were performed according to the following groups:

1. Vehicle (this sample was not treated in the 2^nd^ VDT)
2. Treated with 2ME2 (250 µM) for 1 VDT
3. Treated simultaneously with 2ME2 (250 µM) and IUdR (1 µM) for 1 VDT
4. Treated with 2ME2 (250 µM) for 1 VDT then irradiated with ^60^Co (2 Gy)
5. Treated simultaneously with 2ME2 (250 µM) and IUdR (1 µM) for 1 VDT then irradiated with ^60^Co (2 Gy)

Then the DNA damage was evaluated using the alkaline comet assay method.

**Results:**

The results showed that 2ME2 in combination with gamma irradiation of ^60^Co significantly (p<0.001) increased the DNA damage by IUdR as compared to the control group. Thus the combination of these two agents increased the cytogenetic effects of IUdR in the spheroid culture model of U87MG glioblastoma cell lines.

**Conclusion:**

By inhibiting the HIF-1α protein and preventing the G_0_ phase arrest, 2ME2 causes an increased progression into S phase and increases the IUdR absorption. Then the DNA damage in the spheroid cells increases as the uptake of IUdR is increased. These results suggest that the combined use of 2ME2 and ^60^Co can increase the radiosensitization effect of IUdR.

## introduction

Gliomas are the most common central nervous system tumors and the glioblastoma multiforme (GBM) is the most common primary brain tumor in adults as well as one of the most aggressive cancers in man (1). In 2003, 18300 cases of malignant glioma and 13100 deaths due to this disease were reported in the USA. The malignant glioma is often treated via surgery followed by radiation (2-5). Unfortunately, the irradiation effective enough to control the tumors far exceeds the tolerance of normal brain tissues (6). Thus, to avoid such unfavorable outcomes; methods which sensitize the tumor cells to ionizing radiation (IR) are used. Iododeoxyuridine (IUdR) is a known radiosensitizer that selectively affects the cells.

IUdR is a halogenated thymidine analogue, which incorporates into DNA instead of thymine during DNA replication and increases the radiosensitization of cells. The process of IUdR radiosensitization is totally unexplained; however it is wellknown that DNA damage caused by single and double strand breaks are increased in the presence of IUdR (7). IUdR is activated in the synthesis phase (7); therefore using IUdR when the tumor size is increased and the cells in the median layers suffer from hypoxia due to oxygen deficiency, means IUdR cannot incorporates into DNA.

Hypoxia induces cell cycle arrest in the G_0_ phase (8). In this condition, the IUdR absorption is significantly reduced (9). An important component of the hypoxic response is the activation of the hypoxia inducible factor 1 (HIF-1) transcription factor. Enhancement of this protein level leads to cell cycle arrest (10). Under normoxic conditions, HIF-1α has a short half lifetime (t_1∕2_=0.5 minute) and degrades rapidly (11). Under hypoxia conditions, HIF-1α is transferred from cytoplasm to nucleus and by attaching to HIF-1β, forms the HIF-1 complex (12, 13).

The activity of HIF-1 complex depends on the interaction between hypoxia response elements (HREs) and HIF-1α (14). This interaction activates more than 60 genes with different functions, leading to an increase in O_2_ delivery (15). These genes include erythropoietine (EPO), glucose transporters, glycolytic enzymes and vascular endothelial growth factor (VEGF) (16). Hypoxia increases the expression of EPO, which is required for the formation of red blood cells. An increase in the number of erythrocytes enhances the delivery of oxygen to tissues (17). Angiogenesis is the result of VEGF synthesis in the hypoxia condition, which itself leads into an increase in vascular density and consequently a reduction of the oxygen diffusion distance (18-20).

Research shows that 2-Methoxystradiol (2ME2) inhibits activation of HIF-1α in the hypoxia condition (21). 2ME2 is an estrogen metabolite that inhibits the proliferation, migration and invasion of the endothelial cell (21, 22). Recent studies show that 2ME2 inhibits HIF-1α by depolymerizing the microtubule (23); however this process is still unexplained. The HIF-1α inhibition by 2ME2 is caused by a reduction in the HIF-1α protein levels. The decrease in the HIF-1α levels is accomplished by either reducing the synthesis or increasing the degradation of this protein or both (24). The advantage of 2ME2 over the other drugs that inhibit HIF-1α is that unlike other drugs, 2ME2 is not toxic and does not have the side effects of those drugs. The low toxicity of 2ME2 can be partially due to its fast reversibility (25).

The radiosesitization of most of the glioma cells in the monolayer culture is a very weak reflection of tumor behavior (26). Cells in the spheroid model, similar to the real tumors, are generally more radioresistant than the monolayer model. Spheroids are a three-dimensional form of cell, which have been accepted as an in-vitro model of a solid tumor (27). The absorption of IUdR decreases with the increase in the diameter of the spheroid (28). Research shows that the monolayer SQ5 cells do not express the HIF-1 protein. In contrast, the spheroid and xenograft cells show higher expressions of HIF-1. This finding suggests that HIF-1 expression is enhanced during the growth of threedimensional cell structures (29).

For more than two decades, the comet assay or single- cell gel electrophoresis (SCGE) has been one of the standard methods for the assessment DNA damage (30). This technique is based on the detection of DNA strand breaks in the single cells (31).

Damage is quantified as comet tail moment, which represents the extent of DNA damage in individual cells (32). The comet assay is also a precise and appropriate method for evaluating cell death based on DNA damage in spheroid cultures (33).

In the present study, we have investigated the combined effect of 2ME2 and ^60^Co on the level of induced DNA damage caused by IUdR in the spheroid model of the U87MG glioblastoma cell line. U87MG is an established cell line that can self-assemble into large, stable spheroids through a combination of intracellular communication and diffusion. In this study, we used spheroids with 350 µm diameters. This guarantees the existence of hypoxic cells.

## Materials and Methods

### Cell line

Human glioblastoma cell line U87MG was purchased from the Pasteur Institute of Iran. This cell line was cultured in Minimum Essential Medium (MEM) (Gibco) containing 10% fetal bovine serum (FBS) (Biosera), 100 U/ml of penicillin and 100 mg/ml of streptomycin (Biosera).

### Monolayer cultureA

Cells were cultured as a monolayer at a density of 10^4^ cells/cm^2^ in T-25 tissue culture flasks (NUNC). Cultures were maintained at 37℃ in a humidified atmosphere and 5% CO_2_. Cells were harvested by trypsinizing cultures with 0.25% trypsin and 0.03% ethylenediaminetetraacetic acid (EDTA) (Sigma) in phosphate buffer saline (PBS).

### Spheroid culture

Spheroids were cultured using the liquid overlay technique. 5 × 10^5^ cells were seeded into 100 mm petridishes (Greiner) coated with a thin layer of 1% agar with 10 ml of MEM supplemented with 10% FBS. The plates were incubated at 37℃ in a humidified atmosphere and 5% CO_2_. Half of the culture medium was replaced with fresh culture medium every three days.

### IGrowth curve

After three passages of monolayer culture, Cells were cultured at a density of 10000 per well in multiwell plates (24 wells/plate) (Greiner). The multiwell was incubated at 37℃ in a humidified atmosphere and 5% CO_2_. For nine days, at 24-hour intervals, the cells from triplicate wells were removed by 1mM EDTA/0.25% trypsin (w/v) treatment and counted in a hemocytometer. An average of nine counts was used to define each point (Mean ± SEM). Half of the culture medium was replaced with fresh medium twice per week. Then the growth curve was plotted. In the linear area or logarithmic phase of the curve, the cells follow this equation: N=N_0_ × e^bt^ Here N_0_ is the initial number of the cells, N is the number of the cells after time t, and b shows the gradient of the logarithmic phase of the curve. Then, the population doubling time of the cells is determined according to the gradient of the logarithmic phase of the curve.

### Drug treatment and Gamma radiation

U87MG cells were cultured as spheroids with 350 µm diameters. As control, the spheroids on one plate were not treated. Other cultures were pretreated with 2ME2 (250µM) for one 1 VDT. After this time, the subsequent treatments were performed according to the following groups:

1. Vehicle (this sample was not treated in the 2^nd^ VDT)
2. Treated with 2ME2 (250 µM) for 1 VDT
3. Treated simultaneously with 2ME2 (250 µM) and IUdR (1 µM) for 1 VDT
4. Treated with 2ME2 (250 µM) for 1 VDT then irradiated with ^60^Co (2 Gy) (34)
5. Treated simultaneously with 2ME2 (250 µM) and IUdR (1 µM) for 1 VDT then irradiated with ^60^Co (2 Gy)

Then the DNA damage was evaluated using the alkaline comet assay method.

### Trypan blue exclusion assay

A suspension of treated and control single cells from spheroid cultures were mixed with trypan blue at a ratio of 9:1. After a few minutes the mixture was examined under a light microscope (Leica, DMLS), and the blue cells were considered dead. The percentage of unstained cells out of the total number of cells was the viability of each cell category.

### Comet assay

**Fig 1:**
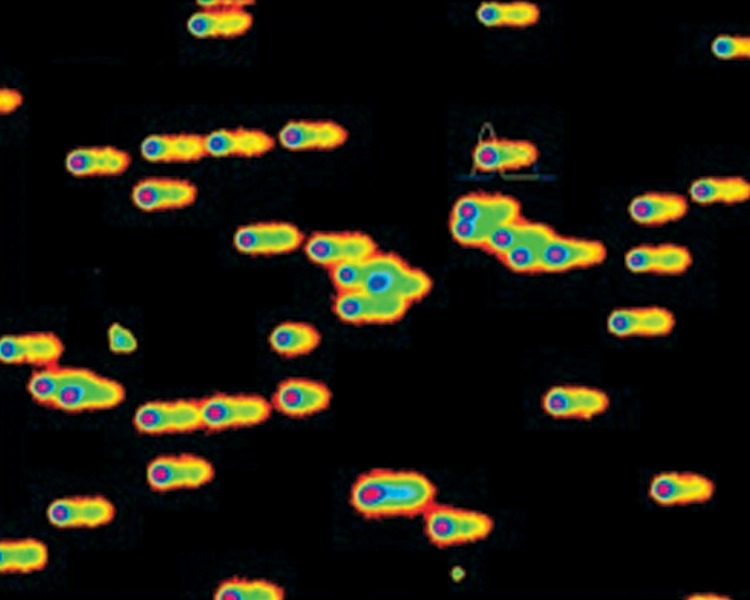
Capture of an image from the microscope camera using Comet Score software

The induction of DNA damage due to 2ME2 alone or in combination with IUdR and ^60^Co was determined by alkaline comet assay in U87MG spheroid cells. The alkaline comet assay in this study was a modification of the method described by Singh et al. (35). Ordinary microscope slides were coated with 1% normal melting point agarose (Merck). The treated and control cells were counted in a hemocytometer (36) and approximately 10,000 cells in 10 µl PBS were suspended in 100 µL of 0.5% low melting point agarose (Merck). The cell suspension was rapidly pipetted onto the first agarose layer. The slides were allowed to solidify, then immersed in freshly prepared lysis buffer (2.5 M NaCl, 100 mM EDTA, 10 mM Tris-base with 1% Triton X-100, pH=10) and incubated for an hour. From that point on, all the steps were performed at 4℃. The slides were removed from the lysis buffer and placed in a horizontal gel electrophoresis tank (Cleaver Scientific Ltd, CSL-COM20) which was filled with fresh cold denaturation buffer (300 mM NaOH, 1mM EDTA, pH=13). The slides were left in the solution for 30 minutes. Electrophoresis was conducted in the same denaturation buffer for 30 minutes using 1V/cm voltage and a current of 300 mA. Following electrophoresis, the slides were washed in Tris buffer (0.4 M Tris-HCl, pH=7.5) to neutralize the excess alkali. Finally, the slides were stained with ethidium bromide (20 µg/mL). The individual cells or comets were viewed and photographed using a fluorescent microscope (Zeiss, Axioskop 2 plus) equipped with an ethidium bromide filter (excitation filter, 535 nm; emission filter, 610 nm) and a CCD camera (Hitachi, KP-D20BP). The photographs were analyzed using Comet Score® software. Figure 1 shows the capture of an image from the microscope camera using Comet Score software.

### Evaluation of DNA damage

A total of 100 individual cells on each slide and three slides for each sample were scored visually as belonging to one of five predefined classes according to tail length, and given a value of 0, 1, 2, 3, or 4 (from no tailing, 0, to maximally tailing, 4).

The total score for comets could range from 0 (all no tailing) to 400 (all maximally tailing).

DD (au) = (0_n0_ + 1_n1_+ 2_n2_+ 3_n3_ + 4_n4_) ∕ (Σn ∕100)

Where DD (au) is the arbitrary unit DNA damage score, n0-n4 is the number of class 0-4 comets, and Σn is the total number of scored comets. Coefficients 0-4 are weighting factors for each class of comet (37, 38). One may suspect that the visual classification may be inferior to computerized analyses, such as tail moment analysis of images captured by CCD camera. DNA damage was quantified as an increase in tail moment, the product of the amount of DNA (fluorescence) in the tail, and the distance between the means of the head and tail fluorescence distributions.

### Statistical analysis

Data were given as mean ± SEM, with 'n' denoting the number of experiments. Statistical analysis was performed using one-way analysis of variance (ANOVA) followed by Tukey's test as the posthoc analysis using SPSS version 12. The value of p<0.05 was considered to be significant.

## Results

### Cell characteristics

#### Monolayer culture

**Fig 2:**
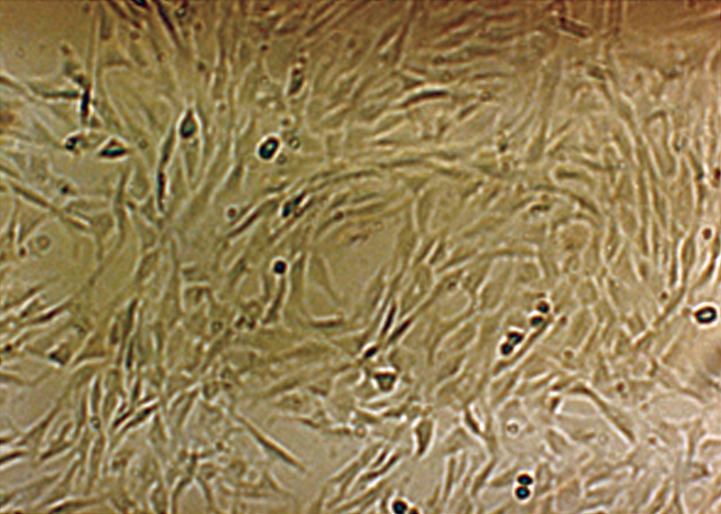
Phase contrast micrograph of U87MG cells in the monolayer culture with ×10 magnification

**Fig 3:**
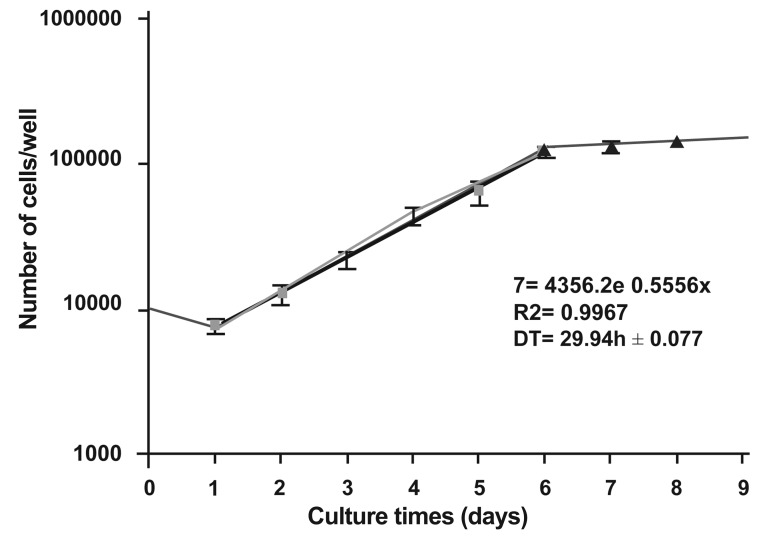
Growth curve of **U87MG** cell line in the monolayer cultures. An average of nine counts was used to define each point. **Mean ± SEM** of three experiments.

The U87MG glioblastoma cell line grows as a monolayer on tissue culture flasks. Figure 2 shows the phase contrast micrographs of the monolayer culture of the U87MG cell line. The growth curve of these cells in the monolayer culture is shown in figure 3. The population doubling time calculated from this curve was approximately 29.94 hours.

#### Spheroid culture

**fig 4:**
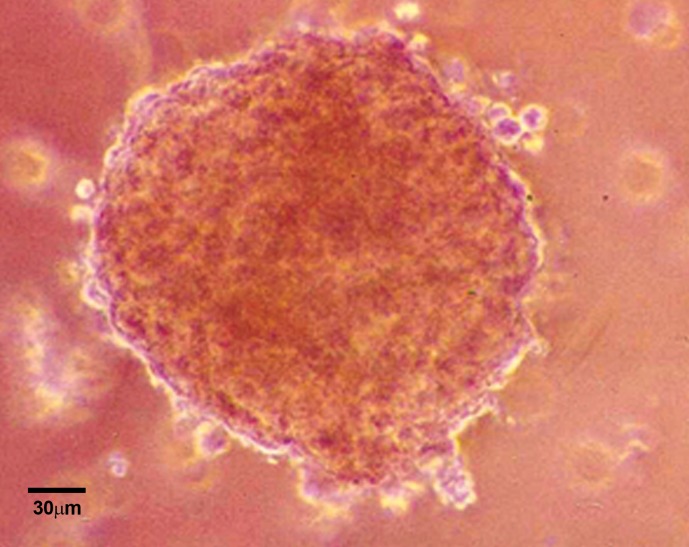
Phase contrast micrograph of **U87MG** cell spheroid with 350 µm diameter on day 24 after culture initiation. Magnification is ×10

The U87MG cells could form spheroids in liquid overlay cultures. Figure 4 shows the phase contrast micrograph of these spheroids with 350 µm diameters 24 days after culture initiation. At this time, spheroids had formed completely into well-rounded structures composed of numerous highly compact cells in which it was difficult to distinguish individual cells from each other (39). In general, the formation time of spheroids depends on the initial number of cells plated. For instance, when 5x105 cells were plated in the 100 mm petridishes on a thin layer of agar, the spheroids were formed within two to three days. The volume doubling time of these spheroids is approximately 67 hours (34), which was applied as the drug treatment time. The comet assay was used for the evaluation of DNA damage after the drug treatment and radiation.

### DNA damage

**Fig 5:**
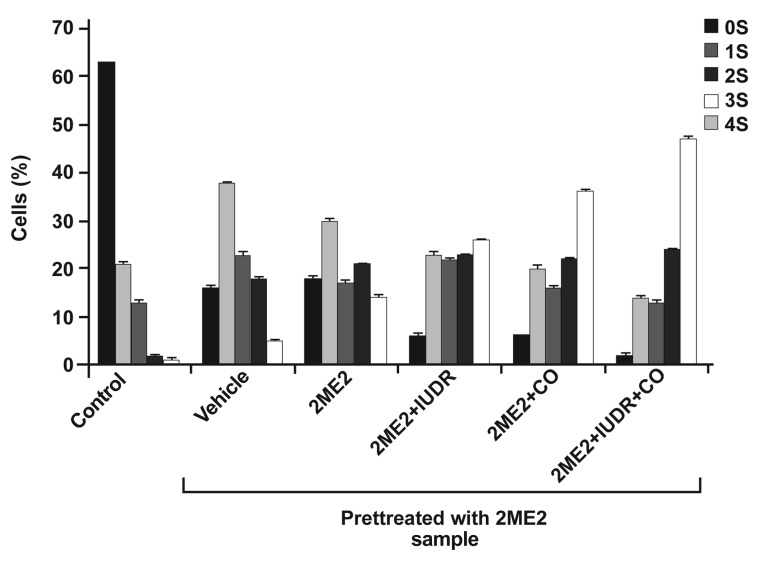
Distribution of **DNA** migrations (stages 0 to 4) among **U87MG** cells of 350 µm spheroids after pretreatment for 67 hours (one volume doubling time) with **250 µM 2ME2** and treatment for the next volume doubling time with **2ME2**, **IUdR** and ^60^Co gamma radiation. Data based on the analysis of 100 cells per slide, triplicate slides per samples.

**Fig 6:**
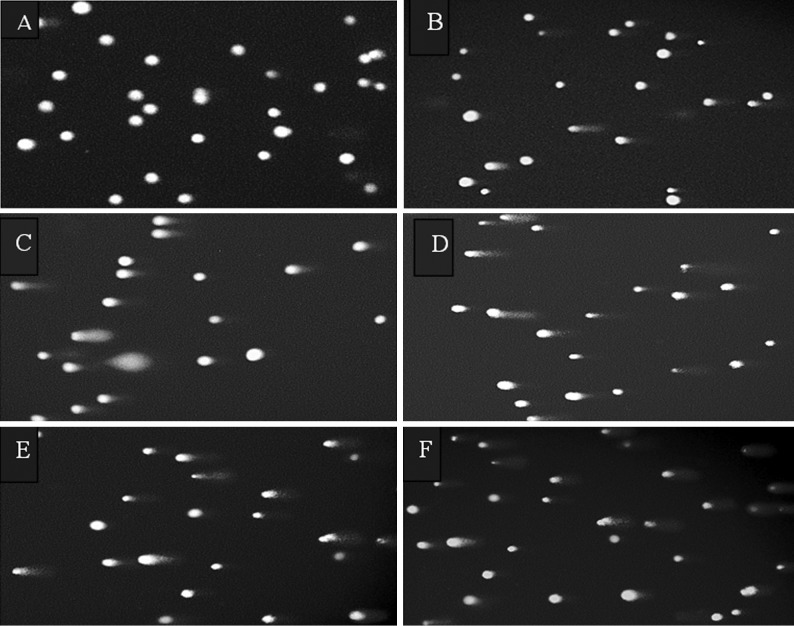
Images of single cell gel electrophoresis (comet assay) of **U87MG** cells of 350 µm spheroids after pretreatment for 67 hours (one volume doubling time) with **250 µM 2ME2** and treatment for the next volume doubling time with **2ME2, IUdR** and ^60^Co gamma radiation. Samples as follows: **A.** control, samples **B** to **F** were pretreated with **250µM 2ME2** and then treated as follows: **B.** vehicle, **C.** **2ME2, D. 2ME2 + IUdR, E. 2ME2 + ^60^Co, F. 2ME2 + IUdR + ^60^Co.**

Alkaline comet assays were used for the evaluation of DNA damage. Figure 5 shows the intercellular distribution of DNA migration (number of cells in the five visual comet classes) among control and treated cells. We observed a significant increase in the number of comets scored in the visual class with the combination treatment of 2ME2 + IUdR + irradiation of ^60^Co. Exposure to 2ME2 + IUdR + irradiation of ^60^Co revealed that the majority of comets were progressively distributed to the next visual category of higher DNA damage. Figure 6 shows the images of single cell gel electrophoresis (comet assay) of U87MG cells of 350 µm spheroids after pretreatment for 67 hours (one volume doubling time) with 250 µM 2ME2 and treatment for the next volume doubling time with 2ME2, IUdR and ^60^C gamma radiation.

**Fig 7:**
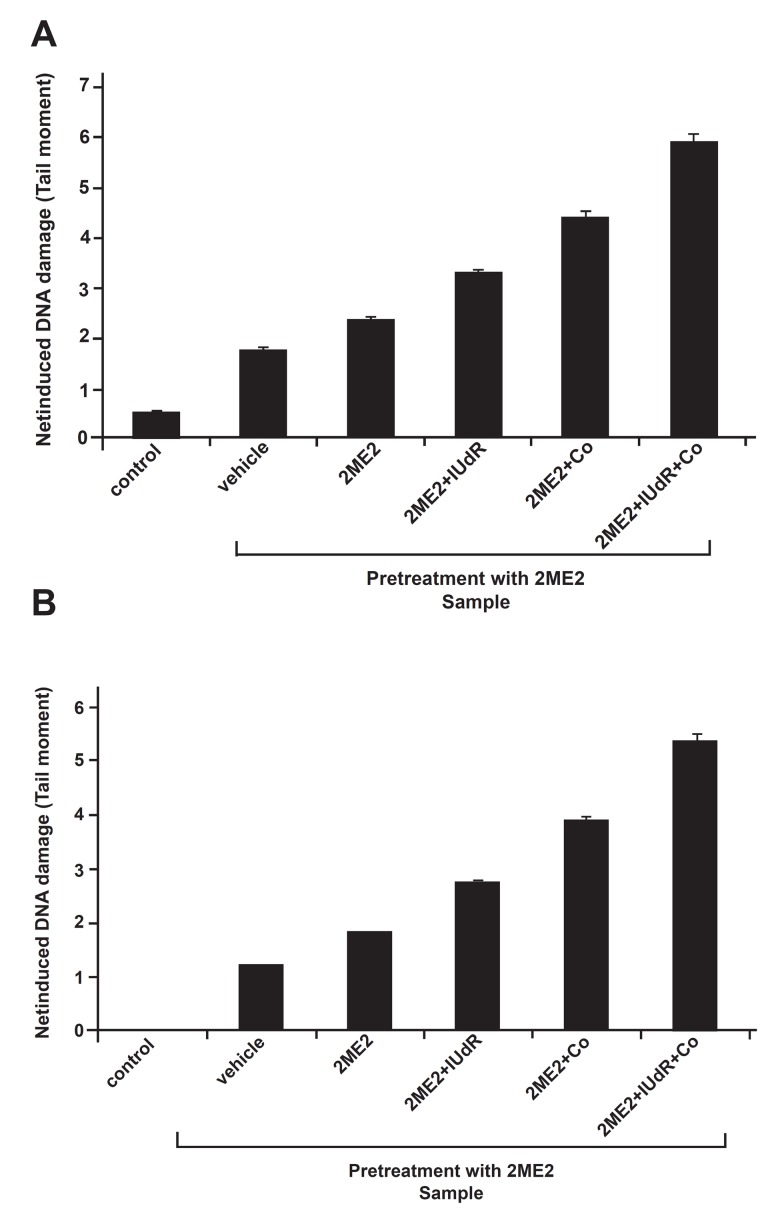
The effects of drugs and radiation on **A**) induced DNA strand breaks **(DD_0_)** and **B**) net induced **DNA** strand breaks **(DD - DD_0_)** of **U87MG** cells from spheroid cultures. Single cells were analyzed for **DNA** single strand breaks. Tail moment, an indication of **DNA** strand breakage, was measured using the alkaline comet assay. **Means ± SEM** of three experiments.

The average tail moments in each category of cells was used as an indication of DNA damage. Table 1A, B and figure 7A, B show quantitative measurements of DNA damage by the comet score program. They show respectively the induced DNA damage (DD0) and the net induced DNA damage (DD-DD0). As can be seen in both figures and tables, 2ME2 can significantly increase the DNA damage (p<0.001). The extent of damage in the 2ME2 group is significantly more than in the vehicle group (p<0.001). In other words, with the increase of incubation time from 1 VDT to 2 VDT in pretreated 2ME2 spheroids, DNA damage increases in the cells. Moreover, simultaneous treatment of cells with 2ME2 and IUdR can significantly increase the tail moment as compared to 2ME2 (p<0.001), as shown in the comparison of 2ME2 + ^60^Co with the 2ME2 group. Furthermore, the DNA damage significantly increased in the presence of 2ME2 + IUdR + irradiation of ^60^Co as compared to the two groups of 2ME2 + IUdR and 2ME2 + ^60^Co (p<0.001).

Table 2 shows the increasing DNA damage percentage in 350 µm spheroids in the three groups of 2ME2/IUdR, 2ME2/^60^Co and 2ME2/IUdR/^60^Co in comparison with the group of 2ME2. As can be seen, the effect of combined treatment with 2ME2/ IUdR/^60^Co is greater than the sum of the effects of the two groups of IUdR/2ME2 and ^60^Co/2ME2.

Table 1The effects of drugs and radiation on **A**) induced DNA strand breaks **(DD_0_)** and **B**) net induced **DNA** strand breaks **(DD - DD_0_)** of **U87MG** cells from spheroid cultures. Single cells were analyzed for **DNA** single strand breaks. Tail moment, an indication of **DNA** strand breakage, was measured using the alkaline comet assay. **Means ± SEM** of three experiments.

**A:** 


Group			Tail moment ± SE
Pretreatment (1^st^ VDT)		Treatment (2^nd^ VDT)	
2ME2	Control		0.518 ± 0.047
		Vhicle	1.768 ± 0.057
		2ME2	2.366 ± 0.065
		2ME2+IUdR	3.296 ± 0.072
		2ME2+Co	4.418 ± 0.124
		2ME2+IUdR+Co	5.907 ± 0.162

**B:** 


Group			Tail moment ± SE
Pretreatment (1^st^ VDT)		Treatment (2^nd^ VDT)	
2ME2	Control		0 ± 0
		Vhicle	1.249 ± 0.018
		2ME2	1.848 ± 0.016
		2ME2+IUdR	2.778 ± 0.025
		2ME2+Co	3.899 ± 0.107
		2ME2+IUdR+Co	5.389 ± 0.151

**Table 2:** Increases in **DNA** damage percentages in **U87MG** spheroids in three groups of **2ME2/IUdR**, **2ME2/^60^Co** and **2ME2/IUdR/^60^Co** in comparison with the group of **2ME2**


Increase in DNA damage percentage in group of 2ME2/ IUdR in comparison with 2ME2	Increase in DNA damage percentage in group of 2ME2/^60^Co in comparison with 2ME2	Increase in DNA damage percentage in group of 2ME2/ IUdR/^60^Co in comparison with 2ME2
37.5%	83%	145%


## Discussion

IUdR is a halogenated thymidine analogue which incorporates into DNA instead of thymidine during DNA replication and increases the radiosensitization of the cells (7). When the tumor size is increased, the cells in the median layers suffer from hypoxia due to oxygen deficiency, and the cells respond to hypoxia through the G_0_ arrest (8). In this condition, IUdR absorption is significantly reduced (9). HIF-1α is the key regulatory element of the hypoxic response of cells. Enhancement of this protein level causes an increased progression into the G_0_ phase (10).

The best-known molecular process, which is necessary for the G1/S phase transition, is retinoblastoma (RB) phosphorylation. Studies show that the arrest in the cell cycle by hypoxia in the G1 phase depends on the decrease in CDK activity. The CDK activity can be inhibited by cycline dependent kinase inhibitors (CDKIs) such as p21 and p27. These inhibitors cause RB hypophosphorylation and consequently promote a G1 arrest (40-42). 2-Methoxystradiol can inhibit HIF-1α expression and prevent this protein’s activity in hypoxia (21). 2ME2 is an estrogen metabolite that inhibits the proliferation, migration and endothelial cell invasion (21, 22). Although 2ME2 is an estrogen metabolite, it has low affinity to estrogen receptors and its antiproliferation activity is independent of the estrogen receptor interaction (43). Recent studies have shown that 2ME2 inhibits HIF-1α by depolymerizing microtubules (23). 2ME2 binds to the colchicine-binding site of tubulin (a site that is at the α/β tubulin interface near α tubulin) and disrupts lateral contacts between protofilaments, which leads to microtubule depolymerization (44). It has been suggested that some physiological differences may exist between cell growth in two-dimensional cultures (monolayer cultures) and multicellular tumor spheroids (44-46). A research conducted on the growth of human glioma cells in these two systems showed different degrees of sensitivity to radioionated IUdR (47). Several authors have reported a higher radioresistance of cells in spheroids compared with monolayer cultures. The radioresistance of spheroid cultures is attributed to the hypoxic cells in the median layer of the spheroid (48-51).

In the present study, we have examined IUdR radiosensitization combined with 2ME2 in spheroid cultures of human glioblastoma cell line U87MG. This experiment was performed with 350µm diameter spheroids. This guarantees the existence of the hypoxic and G_0_ cells. Our previous studies showed that IUdR significantly increases cell damage compared to the control group and as a radiosensitizer it can increase radiationinduced DNA strand breaks (34). Our results reveal that 2ME2 pretreatment significantly increases the cell damage compared to the control group.

The ability of 2ME2 to induce damage and prevent tumor growth correlates with its antitumor effects. The antitumor effects of 2ME2 on cancer cells involve the activation of apoptotic cascades. 2ME2 is able to initiate apoptosis by different pathways such as the activation of cell surface death receptors and the mitochondrial apoptotic pathway (21). The present study revealed that 2ME2 inhibits proliferation and promotes apoptosis of glioma cells. Moreover, increasing the incubation time from 1 VDT to 2 VDT in pretreated 2ME2 cells leads to the enhancement of cell damage. Due to an increase in the spheroid size, the hypoxic cells in the median layers of the spheroid, as well as the HIF- 1α protein expression, increase.

Our hypothesis is that 2ME2 treatment in the second VDT prevents the new HIF-1α protein expression and suppresses the activity of previous HIF-1α proteins, consequently enhancing the DNA damage. In addition, the cell treatment with 2ME2 and IUdR simultaneously increases the cell damage before and after radiation. These results show that using 2ME2 in glioma cells can increase the cell damage induced by the IUdR radiosensitizer significantly. The reason for this is 2ME2 inhibiting the HIF-1α protein. By suppressing the activity and expression of HIF-1α, 2ME2 causes an increased progression into S phase and increases the IUdR absorption. Then the enhanced absorption of IUdR leads to increased damage of DNA. The inhibition of HIF-1α by 2ME2 is due to the decrease in HIF-1α protein levels, which is a result of either the protein synthesis reduction or the increase in protein degradation, or both. Furthermore, the DNA damage is greater in the presence of 2ME2 when the cells are irradiated by ^60^Co, compared to treatment with IUdR. This could be due to an increase in the extent of damage in irradiated cells. The types of damage include exchanging in organic bases and sugar components of DNA, as well as the inducement of DNA single and double strand breaks (52).

## Conclusion

Combined treatment with 2ME2 and ^60^Co significantly increased the damage caused by IUdR. Our findings support the pretreatment of cells with 2ME2/IUdR before irradiation with ^60^Co to enhance tumor radiosensitization and possibly improve the therapeutic index for radiation. Our purpose for further studies is to make use of a carrier such as nanoparticles to increase delivery of IUdR into cells and its uptake into the DNA, and then evaluate the combined effects of these agents on the cells.

## References

[1] Krakstad C, Chekenya M (2010). Survival signalling and apoptosis
resistance in glioblastomas: opportunities for targeted
therapeutics. Mol Cancer.

[2] Walker MD, Alexander E Jr, Hunt WE, MacCarty CS, Mahaley MS Jr, Mealey J Jr (1978). Evaluation of BCNU and/or
radiotherapy in the treatment of anaplastic gliomas. A cooperative
clinical trial. J Neurosurg.

[3] Walker MD, Green SB, Byar DP, Alexander E Jr, Batzdorf U, Brooks WH (1980). Randomized comparisons of radiotherapy
and nitrosoureas for the treatment of malignant glioma after
surgery. N Engl J Med.

[4] Green SB, Byar DP, Walker MD, Pistenmaa DA, Alexander E Jr, Batzdorf U (1983). Comparisons of carmustine,
procarbazine, and high-dose methylprednisolone as additions
to surgery and radiotherapy for the treatment of malignant
glioma. Cancer Treat Rep.

[5] Jemal A, Murray T, Samuels A, Ghafoor A, Ward E, Thun MJ (2003). Cancer statistics, 2003. CA Cancer J Clin.

[6] Sheline GE, Wara WM Smith V (1980). Therapeutic irradiation and brain
injury. Int J Radiat Oncol Biol Phys.

[7] Aziz MA, JE Schupp J E, Kinsella TJ (2009). Modulation of the
activity of methyl binding domain protein 4 (MBD4/MED1)
while processing iododeoxyuridine generated DNA mispairs. Cancer Biol Ther.

[8] Freyer JP, Tustanoff E, Franko AJ, Sutherland RM (1984). In situ
oxygen consumption rates of cells in V-79 multicellular spheroids
during growth. J Cell Physiol.

[9] Wijffels KI, Marres HA, Peters JP, Rijken PF, van der Kogel AJ, Kaanders JH (2008). Tumour cell proliferation under hypoxic
conditions in human head and neck squamous cell
carcinomas. Oral Oncol.

[10] Q Ke, Costa M (2006). Hypoxia-inducible factor-1 (HIF-1). Mol Pharmacol.

[11] Salceda S, Caro J (1997). Hypoxia-inducible factor 1alpha
(HIF-1alpha) protein is rapidly degraded by the ubiquitin-proteasome
system under normoxic conditions. Its stabilization
by hypoxia depends on redox-induced changes. J Biol Chem.

[12] Huang LE, Arany Z, Livingston DM, Bunn HF (1996). Activation
of hypoxia-inducible transcription factor depends primarily
upon redox-sensitive stabilization of its alpha subunit. J Biol Chem.

[13] Kallio PJ, Pongratz I, Gradin K, McGuire J, Poellinger L (1997). Activation of hypoxia-inducible factor 1alpha: posttranscriptional
regulation and conformational change by recruitment
of the Arnt transcription factor. Proc Natl Acad Sci USA.

[14] Lando D, Peet DJ, Whelan DA, Gorman JJ, Whitelaw ML (2002). Asparagine hydroxylation of the HIF transactivation domain
a hypoxic switch. Science.

[15] Iida T, Mine S, Fujimoto H, Suzuki K, Minami Y, Tanaka Y (2002). Hypoxia-inducible factor-1alpha induces cell cycle arrest
of endothelial cells. Genes Cells.

[16] Chen L, Endler A, Shibasaki F (2009). Hypoxia and angiogenesis:
regulation of hypoxia-inducible factors via novel binding
factors. Exp Mol Med.

[17] Semenza GL, Nejfelt MK, Chi SM, Antonarakis SE (1991). Hypoxia-
inducible nuclear factors bind to an enhancer element
located 3' to the human erythropoietin gene. Proc Natl Acad Sci U S A.

[18] Conway EM, Collen D, Carmeliet P (2001). Molecular mechanisms
of blood vessel growth. Cardiovasc Res.

[19] Josko J, GwÓźdź B, Jedrzejowska-Szypułka H, Hendryk S (2000). Vascular endothelial growth factor (VEGF) and its effect
on angiogenesis. Med Sci Monit.

[20] Semenza GL (2009). Involvement of oxygen-sensing pathways
in physiologic and pathologic erythropoiesis. Blood.

[21] Becker CM, Rohwer N, Funakoshi T, Cramer T, Bernhardt W, Birsner A (2008). 2-methoxyestradiol inhibits hypoxia-inducible
factor-1{alpha} and suppresses growth of lesions in a mouse
model of endometriosis. Am J Pathol.

[22] Lakhani NJ, Sparreboom A, Xu X, Veenstra TD, Venitz J, Dahut WL (2007). Characterization of in vitro and in vivo
metabolic pathways of the investigational anticancer agent,
2-methoxyestradiol. J Pharm Sci.

[23] Mabjeesh NJ, Escuin D, LaVallee TM, Pribluda VS, Swartz GM, Johnson MS (2003). 2ME2 inhibits tumor growth and
angiogenesis by disrupting microtubules and dysregulating
HIF. Cancer Cell.

[24] Semenza GL (2007). Evaluation of HIF-1 inhibitors as anticancer
agents. Drug Discov Today.

[25] Kamath K, Okouneva T, Larson G, Panda D, Wilson L, Jordan MA (2006). 2-Methoxyestradiol suppresses microtubule
dynamics and arrests mitosis without depolymerizing microtubules. Mol Cancer Ther.

[26] Taghian A, Ramsay J, Allalunis-Turner J, Budach W, Gioioso D, Pardo F (1993). Intrinsic radiation sensitivity may
not be the major determinant of the poor clinical outcome
of glioblastoma multiforme. Int J Radiat Oncol Biol Phys.

[27] Fazeli GR, Khoei S, Nikoofar AR, Goliaei B (2007). DNA damage in
tumor spheroids compared to monolayer cultures exposed
to ionizing radiation. Iranian Journal of Radiation Research.

[28] Freyer JP (1998). Decreased mitochondrial function in quiescent
cells isolated from multicellular tumor spheroids. J Cell Physiol.

[29] Zou Y, Cheng C, Omura-Minamisawa M, Kang Y, Hara T, Guan X (2010). The suppression of hypoxia-inducible factor
and vascular endothelial growth factor by siRNA does
not affect the radiation sensitivity of multicellular tumor
spheroids. J Radiat Res (Tokyo).

[30] Collins AR (2004). The comet assay for DNA damage and repair:
principles, applications, and limitations. Mol Biotechnol.

[31] Olive PL, Banath JP, Durand RE (1990). Heterogeneity in radiation-
induced DNA damage and repair in tumor and normal
cells measured using the "comet" assay. Radiat Res.

[32] Olive PL, Banath JP (1997). Multicell spheroid response to
drugs predicted with the comet assay. Cancer Res.

[33] Olive PL, Vikse CM, Banath JP (1996). Use of the comet assay to
identify cells sensitive to tirapazamine in multicell spheroids
and tumors in mice. Cancer Res.

[34] Neshasteh-Riz A, Saki M, Khoei S (2008). Cytogenetic damages
from Iododeoxyuridine-induced radiosensitivity with and
without Methoxyamine in human glioblastoma spheroids. Yakhteh.

[35] Singh NP, McCoy MT, Tice RR, Schneider E L (1988). A simple
technique for quantitation of low levels of DNA damage in
individual cells. Exp Cell Res.

[36] Macleod KG, Langdon SP, Langdon SP (2004). Essential techniques of cancer
cell culture. Cancer cell culture, methods
and protocols.

[37] Chen CY, Wang YF, Huang WR, Huang YT (2003). Nickel induces
oxidative stress and genotoxicity in human lymphocytes. Toxicol Appl Pharmacol.

[38] Mohseni Meybodi A, Mozdarani H (2009). DNA damage in leukocytes
from Fanconi anemia (FA) patients and heterozygotes
induced by mitomycin C and ionizing radiation
as assessed by the comet and comet-FISH assay. Iran Biomed J.

[39] Khoei S, Goliaei B, Neshasteh-Riz A, Deizadji A (2004). The role
of heat shock protein 70 in the thermoresistance of prostate
cancer cell line spheroids. FEBS Lett.

[40] Gardner LB, Li Q, Park MS, Flanagan WM, Semenza GL, Dang CV (2001). Hypoxia inhibits G1/S transition through regulation
of p27 expression. J Biol Chem.

[41] Krtolica A, Krucher NA, Ludlow JW (1999). Molecular analysis of
selected cell cycle regulatory proteins during aerobic and
hypoxic maintenance of human ovarian carcinoma cells. Br J Cancer.

[42] Sanchez-Puig N, Veprintsev DB, Fersht AR (2005). Binding of
natively unfolded HIF-1alpha ODD domain to p53. Mol Cell.

[43] LaVallee TM, Zhan XH, Herbstritt CJ, Kough EC, Green SJ, Pribluda VS (2002). 2-Methoxyestradiol inhibits proliferation and
induces apoptosis independently of estrogen receptors alpha
and beta. Cancer Res.

[44] Risinger AL, Giles FJ, Mooberry SL (2009). Microtubule dynamics
as a target in oncology. Cancer Treat Rev.

[45] Dobrucki J, Bleehen NM (1985). Cell-cell contact affects cellular sensitivity
to hyperthermia. Br J Cancer.

[46] Wigle JC, Sutherland RM (1985). Increased thermoresistance
developed during growth of small multicellular spheroids. J Cell Physiol.

[47] Neshasteh-Riz A, Mairs RJ, Angerson WJ, Stanton PD, Reeves JR, Rampling R (1998). Differential cytotoxicity of
(123I)IUdR, (125I)IUdR and (131I)IUdR to human glioma
cells in monolayer or spheroid culture: effect of proliferative
heterogeneity and radiation cross-fire. Br J Cancer.

[48] Desoize B, Gimonet D, Jardiller JC (1998). Cell culture as spheroids:
an approach to multicellular resistance. Anticancer Res.

[49] Desoize B, Jardillier J (2000). Multicellular resistance: a
paradigm for clinical resistance?. Crit Rev Oncol
Hematol.

[50] Kerbel RS, Rak J, Kobayashi H, Man MS, St Croix B, Graham CH (1994). Multicellular resistance: a new paradigm to explain
aspects of acquired drug resistance of solid tumors. Cold Spring Harb Symp Quant Biol.

[51] Olive PL, Durand RE (1994). Drug and radiation resistance in
spheroids: cell contact and kinetics. Cancer Metastasis Rev.

[52] Scott SP, Pandita TK (2006). The cellular control of DNA doublestrand
breaks. J Cell Biochem.

